# Rounding up the annual ryegrass genome: High-quality reference genome of *Lolium rigidum*

**DOI:** 10.3389/fgene.2022.1012694

**Published:** 2022-11-01

**Authors:** Jefferson Paril, Gunjan Pandey, Emma M. Barnett, Rahul V. Rane, Leon Court, Thomas Walsh, Alexandre Fournier-Level

**Affiliations:** ^1^ School of BioSciences, University of Melbourne, Parkville, VIC, Australia; ^2^ CSIRO Land and Water, Acton, ACT, Australia; ^3^ CSIRO Health and Biosecurity, Parkville, VIC, Australia

**Keywords:** ryegrass, genome, weed, herbicide, resistance

## Abstract

The genome of the major agricultural weed species, annual ryegrass (*Lolium rigidum*) was assembled, annotated and analysed. Annual ryegrass is a major weed in grain cropping, and has the remarkable capacity to evolve resistance to herbicides with various modes of action. The chromosome-level assembly was achieved using short- and long-read sequencing in combination with Hi-C mapping. The assembly size is 2.44 Gb with N_50_ = 361.79 Mb across 1,764 scaffolds where the seven longest sequences correspond to the seven chromosomes. Genome completeness assessed through BUSCO returned a 99.8% score for complete (unique and duplicated) and fragmented genes using the Viridiplantae set. We found evidence for the expansion of herbicide resistance-related gene families including detoxification genes. The reference genome of *L. rigidum* is a critical asset for leveraging genetic information for the management of this highly problematic weed species.

## Introduction

*Lolium rigidum* (Gaudin and Paschoud 1811) also known as annual ryegrass, rigid ryegrass, or Wimmera grass, is the world’s most herbicide resistant weed species. It has developed resistance to over a dozen different modes of action across a number of herbicides and has the highest incidence of resistance in any weed species (Heap 2022). In particular, it is the first weed species reported to have evolved resistance to glyphosate (Powles et al., 1998).

*L. rigidum* is a diploid grass species with a chromosome number of 2n = 2x = 14 (Terrell 1966; Monaghan 1980) and an estimated genome size of ∼2Gb, similar to that of the closely-related forage crop *Lolium perenne* (Byrne et al., 2015; Frei et al., 2021). This species is known to hybridise with other members of the *Lolium* genus such as *L. multiflorum* and *L. perenne* (Kloot 1983). This genus is thus a complex of cross-compatible species which can produce fertile hybrids and makes species boundaries ambiguous (Naylor 1960; Terrell 1966; Kloot 1983).

*L. rigidum* is a highly-competitive, self-incompatible, wind-pollinated, annual, C3 weed species (Monaghan 1980; Mccraw and Spoor, 1983), which can produce up to 45,000 seeds per square metre in wheat fields where it can achieve high densities (33%–67% abundance in agricultural field conditions; Rerkasem et al., 1980). The combination of its high fecundity and outcrossing reproduction regime results in large and genetically diverse populations with high adaptive potential. The seeds have varying levels of dormancy ensuring their persistence in the soil seedbank (Goggin et al., 2012). Ryegrass infestation causes significant yield reduction in rapeseed and cereal crops (Lemerle et al., 1995) and its seeds can get infected with *Clavibacter toxicus* causing livestock poisoning (Riley and McKay 1991; Ophel et al., 1993).

*L. rigidum* is native to the Mediterranean region and was widely introduced around the world as a pasture crop. In the 19^th^ century, it was introduced to Australia (Kloot 1983) where it successfully adapted through a combination of artificial and natural selection. It is now the major weed in the wheat-growing regions of Australia (Reeves 1976; Medd et al., 1985; Powles and Matthews 1992). We selected a glyphosate-resistant plant from Australia as the source of the reference genome to represent the remarkable capacity of this weed species to evolve resistance to herbicides.

In this paper, we report a reference, chromosomal-level genome assembly of *Lolium rigidum*. This information is a valuable resource towards genomically-informed management of this major agricultural weed species with a particular emphasis on the issue of herbicide resistance evolution.

## Materials and methods

### Plant sampling, tissue culture, and DNA extraction

A single glyphosate-resistant plant from Wagga Wagga (NSW, Australia) was selected as the reference genotype for *Lolium rigidum*. This individual was tissue-cultured to induce embryogenic calli for clonal multiplication and maintenance following the protocol for *Lolium* spp. by Creemers-Molenaar and Beerepoot. (1992). DNA was extracted using Qiagen DNeasy plant mini kit (QIAGEN N.V., Venlo, Netherlands) following manufacturer’s instructions.

### Genome sequencing and assembly

Short- and long-read DNA sequence data were generated and scaffolded using Hi-C sequence information. Short-read sequencing libraries were constructed using NEBNext Ultra II DNA Library Prep kit for Illumina (NEB, United States) and sequenced using HiSeq X platform (Illumina, Inc., San Diego, United States) ran in 150-bp paired-end mode. Adapter sequences were removed from the resulting reads using TrimGalore (v 0.6.6). Long-read sequencing was carried out on MinION (2 libraries using SQK-LSK109 kit and sequenced on FLO-MIN106D flowcell) and PromethION (1 library using SQK-LSK109 kit and sequenced on a FLO-PRO002 flowcell) platforms. Basecalling was performed using *guppy* (v5.1; Wick et al., 2019) under the *dna_r9.4.1_450bps_sup.cfg* model. The long-read sequences were trimmed using *Porechop* (v0.2.4; Wick et al., 2017) and filtered using *filtlong* (v0.2.1) to obtain high quality reads. The long-reads were assembled using *Flye* (v2.9; Kolmogorov and Derek, 2020) with the minimum overlap parameter set to 6,000, kmer size of 17, genome size of 2.25 Gb, and with no scaffolding. Duplicate contigs were purged using *purge_dups* (v1.2.5; Guan et al., 2020) with the default settings. The long-reads were error-corrected and trimmed using *Canu* (v2.2; Koren et al., 2017) under default settings, and used in three rounds of contig polishing using *Racon* (v1.4.22; Vaser et al., 2017) under default settings. This was followed by three rounds of short-read-based polishing using *Polca* (*MaSURCA* v4.0.7; Zimin et al., 2013) to obtain the final contig assembly using default settings. This assembly was assessed using *BUSCO* (v5.2.2; Simão et al., 2015) against the Viridiplantae and Poales lineages’ gene sets (i.e. viriplantae_obd10 and poales_odb10).

A Hi-C library was prepared using 20 mg of leaf tissue and the Arima HiC kit following the manufacturer’s instructions. The library was sequenced on NovaSeq 6000 platform (Illumina, Inc., San Diego, United States) to generate 500 million reads. The final contig assembly was scaffolded based on the genomic topological information using *ALLHiC* (v1; Zhang et al., 2019) and manually curated using JuiceBox (v1.9.8; Dudchenko et al., 2017). The assembly was checked by NCBI’s GenBank decontamination pipeline, and the contaminating sequences were removed. Genome size was estimated based on the kmer distribution of the Illumina sequences with Jellyfish 2.3.0 (Marçais and Kingsford, 2011) and GenomeScope (v1.0.0; Vurture et al., 2017) with kmer ranging from 15 bp to 25 bp and the kmer with the best model fit was used. Genome assembly completeness using k-mer spectrum was automatically assessed during the Polca (*MaSURCA* v4.0.7; Zimin et al., 2013) run. LTR_retriever (Ou et al., 2018) in tandem with GenomeTools (i.e. LTR harvest; Gremme et al., 2013) were used to assess the assembly contiguity.

The assembly is available on the National Center for Biotechnology Information of the United States (NCBI) database under the accession number (SAMN25144995, JAKKIG000000000). Raw Illumina, MinION, PromethION, and Hi-C reads are available under the NCBI Bioproject PRJNA799061. The genome assembly and annotations are available to browse at http://traitnet.adaptive-evolution.org/jbrowse/JBrowse-1.16.11/.

### Transcriptome sequencing, assembly, and genome annotation

Clones from the reference plant established through tissue culture were grown under greenhouse conditions. Two independent samples each of whole seedlings, roots, stems, leaves, inflorescence and meristem tissue were snap-frozen and total RNA was extracted using Isolate II RNA plant kit (Bioline, United Kingdom). RNA sequencing libraries were synthesised for each sample using NEBNext Ultra II stranded RNA library synthesis kits, indexed using the NEBNext Multiplex Oligos for Illumina barcode kit. Libraries were quantified using NEBNext Library Quant KIt for Illumina, normalised, pooled, and sequenced on an Illumina HiSeq X Ten platform to generate ∼257 million 150-bp paired-end reads. The reads were demultiplexed and error-corrected using Rcorrector (v1.0.4; Song and Florea, 2015). Adapters and low quality base pairs were trimmed using TrimGalore (v0.6.0). Ribosomal RNA sequences were discarded when one of the paired-end reads mapped to the sequences present in the SILVA database (v138.1; Quast et al., 2013) using Bowtie2 (v2.3; Langmead & Salzberg, 2012). After filtering, ∼197 million reads were used for *de novo* transcriptome assembly including the rice protein sequences (release 51 Os-Nipponbare-Reference-IRGSP-1.0) as guide and using both Trinity (v2.8.4; Haas et al., 2013) and Oases (v0.2.09; Schulz et al., 2012) as assemblers. The resulting two assemblies were merged into a single compacted meta-assembly using the *De novo* RNA-Seq Assembly Pipeline (Cabau et al., 2017). The filtered reads were re-mapped against the meta-assembly and transcripts with FPKM>1 were included in the transcriptome.

The genome was annotated using NCBI’s genome annotation pipeline using the *de novo* assembled transcriptome. Transposable elements were identified using RepeatMasker and RepeatModeller (v4.1.2 and v2.0.3, respectively; Flynn et al., 2020).

### Comparative genomics

The reference genomes, annotations and coding DNA sequences (CDS) of *Arabidopsis thaliana* (TAIR10 v1), rice (*Oryza sativa*; IRGSP v1), sorghum (*Sorghum bicolor*; NCBI v3), maize (*Zea mays*; B73 Reference NAM v5), and perennial ryegrass (*Lolium perenne*; Kyuss v1) were used for comparative genomics analyses. The well-curated genome of *A. thaliana* was used as the outgroup. Rice and maize genomes represent well-annotated grass genomes. Perennial ryegrass is a closely related species. Sorghum is an additional grass crop species.

OrthoFinder (v2.5.4; Emms and Keyll 2019) was used to cluster all the CDS of the six species into orthogroups which includes paralogs within species and orthologs among species. The resulting orthogroups were assigned to gene families they most likely belong to using HMMER (v3.3.2; Mistry et al., 2013) and PantherHMM gene family models (v17; Mi et al., 2019). Significant gene family contraction and expansion in each of the six genomes were determined using CAFE (v5; De Bie et al., 2006) with a *p*-value<0.01. The significantly expanded gene families were used for gene ontology (GO) enrichment analysis using the GO consortium’s web tool “Gene ontology enrichment analysis tool” (The UniProt Consortium, 2019) with a *p*-value<0.05. An additional GO term enrichment analysis was performed with two random sets of 500 randomly sampled genes from the full set of significantly enriched genes to test the consistency of the analysis result.

Orthogroups consisting of a single gene in each of the six genomes, i.e. single-copy gene orthogroups were used to generate a phylogenetic tree by maximum likelihood. These single-copy gene orthogroups were aligned using MACSE (v2.06; Ranwez et al., 2011) and concatenated into a single alignment. The phylogenetic time tree was generated using IQ-TREE (v2.0.7; Minh et al., 2020), where the best fitting substitution model per orthogroup was selected using ModelFinder (Kalyaanamoorthy et al., 2017), and dates of ancestral nodes were obtained from TimeTree.org (i.e. fossil record estimates of median divergence times between *A. thaliana* and rice estimated to 160 million years ago (MYA), sorghum and perennial ryegrass: 62 MYA, and perennial ryegrass & annual ryegrass: 2.74 MYA).

The rate of transversions at four-fold degenerate sites (4DTv) for each pair of sequences across paralogs within species and orthologs across species was calculated to estimate relative divergence times and identify whole genome duplication (WGD) events. For computational efficiency, only the paralogs and orthologs with 2 to 5 members were included in 4DTv calculations.

### Herbicide resistance genes

The use of a glyphosate-resistant plant as reference genome allowed the investigation of the potential genomic basis of herbicide resistance. The resistance-conferring genomic features may be point mutations in genes coding for essential enzymes targeted by herbicides or in detoxification genes. These mutations can be detected using pairwise rates of synonymous and non-synonymous substitutions, i.e. Ka/Ks ratio (1: neutral, >1:positive selection, <1: stabilising selection), estimated between homologous pairs of protein coding sequences. Additionally, the resistance-conferring changes may be structural variants leading to gene loss or duplication. These can be detected by assessing the patterns of expansion and contraction in the genes coding for the target of glyphosate (i.e. enolpyruvylshikimate phosphate synthase; EPSPS indispensable for aromatic amino acid synthesis), and detoxification-related gene families.

To perform these analyses, 8 enzymes known to confer herbicide resistance were selected: enolpyruvylshikimate phosphate synthase (EPSPS), superoxide dismutase (SOD), ascorbate peroxidase (APX), glutathione S-transferase (GST), monodehydroascorbate reductase (MDAR), glutathione peroxidase (GPX), cytochrome P450 (CYP450), and ATP-binding cassette transporter (ABC). The sequences of these proteins were downloaded from the Universal Protein Resource (UniProt) database. Protein sequences specific to *L. rigidum*, *L. multiflorum*, *A. thaliana*, *O. sativa*, and *Z. mays* were used because of the high quality of the gene annotation in these species. The predicted protein sequences of the six genomes were queried against the herbicide-resistance-related protein sequences and the best matching encoding gene was identified (E-value≤1 ×10^–10^). Significantly expanded and contracted gene families using all six species were identified using CAFE (v5; De Bie et al., 2006) with a *p*-value<0.01. The coding sequences of EPSPS gene paralogs within the annual ryegrass genome and homologs in the other five genomes were further analysed using Ka/Ks ratio across 15-bp non-overlapping sliding windows using KaKs_calculator (version 2; Wang et al., 2009).

The full workflow including the scripts and links to the datasets used for the comparative genomics analysis are found in the README.md file of https://github.com/jeffersonfparil/Lolium_rigidum_genome_assembly_and_annotation.

## Results

### Genome assembly

A total of 294.8 Gb from short- and long-read whole-genome sequencing was generated. Illumina sequencing accounted for 68.69% of this output, Oxford Nanopore sequencing using MinION and Promethion platforms accounted for 31.31%, and the Hi-C library generated 66 Gb of raw sequence data. We estimated the genome size to be 2.26 Gb based on k-mer frequency analysis (kmer = 16 bp found to generate the best model fit according to GenomeScope v1.0.0). The sequencing output information is summarised in Supplementary Table S1.

**FIGURE 1:**
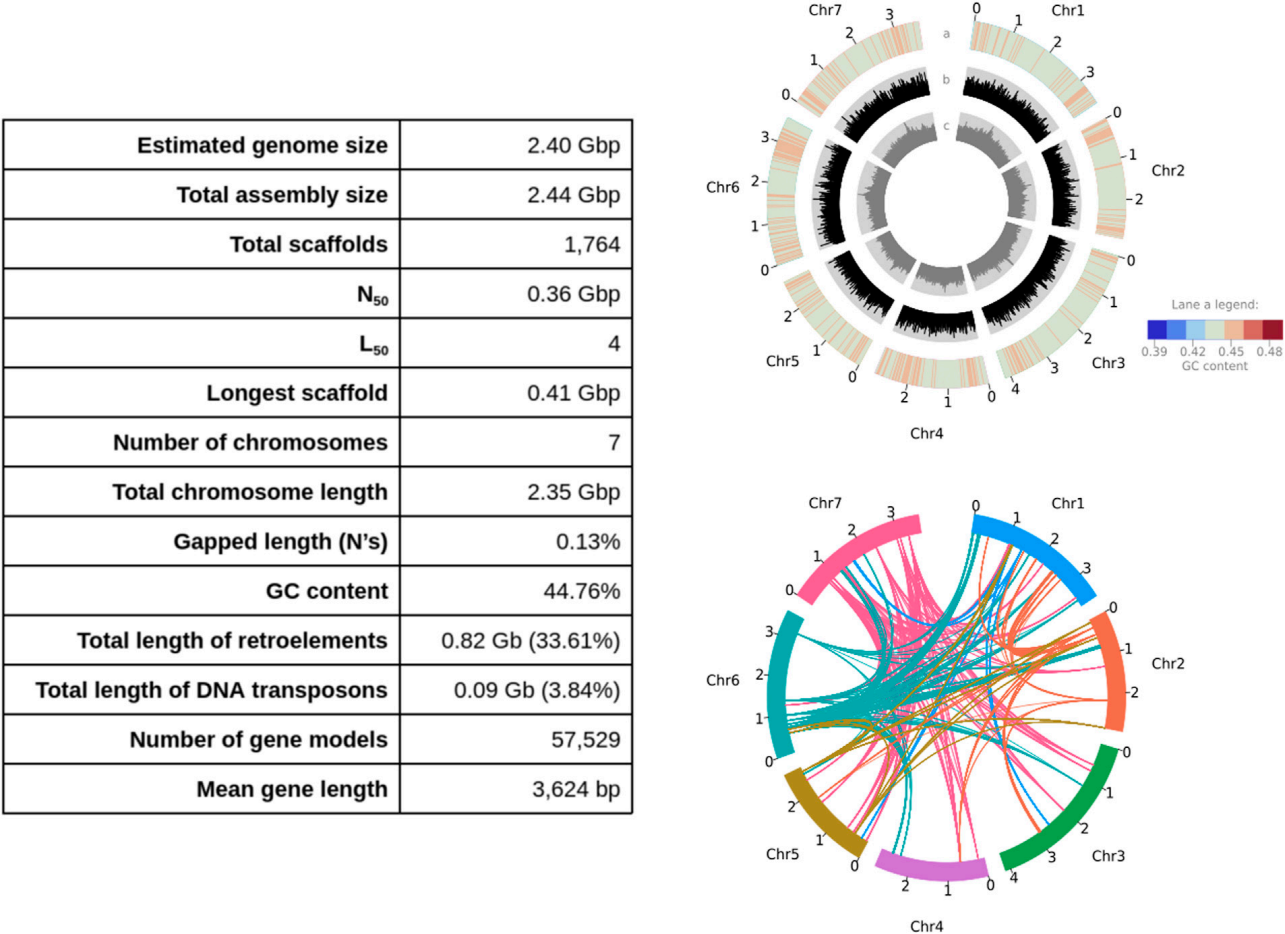
Left panel: Assembly statistics, and right panel: features of the *Lolium rigidum* genome (each tick is ×100 Mb). Top right panel—lane (a) GC content heatmap of mean GC content per 2.35 Mb window (ranging from 42% to 47%); lane (b) distribution of Copia long terminal repeat (LTR) retrotransposon family; lane (c) distribution of Gypsy LTR retrotransposon family. Bottom right panel: chord diagram shows the syntenic relationships within the top 5 orthogroups with the most paralogs in the genome (i.e. F-box domain-containing protein family, F-box protein interaction domain protein-related family, auxin-responsive protein family, and two orthogroups with unknown function: OS06G0725500 and PTHR32141), where the colours match the colours of the chromosome most of the paralogs per orthogroup are located.

The final assembly reached a chromosomal level resolution with a total length 2.44 Gb (N_50_ = 361.79 Mb) over 7 scaffolds and 1,757 unplaced contigs where the 7 scaffolds constitute 96% of the assembly and correspond to the 7 expected chromosomes (Figure 1 left panel and Supplementary Figure S1). This assembly is 99.99% k-mer complete with 39.28 consensus quality score (QV) according to Polca (*MaSURCA* v4.0.7). The average mapping rates of the genomic and transcriptomic sequencing reads to the assembly are 97.48% and 90.92%, respectively. This assembly is 99.8% complete based on BUSCO analysis using the Viridiplantae gene set (i.e. Complete and single-copy [S]: 29.4%, Complete and duplicated [D]: 70.4%, Fragmented [F]: 0.2%, Missing [M]: 0.0%, Total [n]: 425) and 97.2% complete using the Poales gene set (S:32.4%, D:64.8%, F:0.9%, M:1.9%, and n:4,896).

The *L. rigidum* genome assembly mainly consists of interspersed repeats (72.44%), transposable elements and repetitive sequences accounting for 33.61% and 34.99% of the genome, respectively. Among the transposable elements, long terminal repeat (LTR) sequences were predominant (30.91% of the genome), mostly composed of Copia (24.51%) and Gyspy (6.40%) LTRs. The intactness of these LTR retrotransposons (LTR-RTs) which is proportional to the contiguity of the assembly was measured with the LTR assembly index (LAI). The average genome-wide raw LAI was 13.04 (standardised LAI = 7.41) which is above the minimum threshold 2 (0.1% intact LTR-RTs divided by 5% total LTR-RTs; Ou et al., 2018). The distribution of LAI across the genome assembly is shown in Supplementary Figure S2.

### Gene family contraction and expansion

**FIGURE 2:**
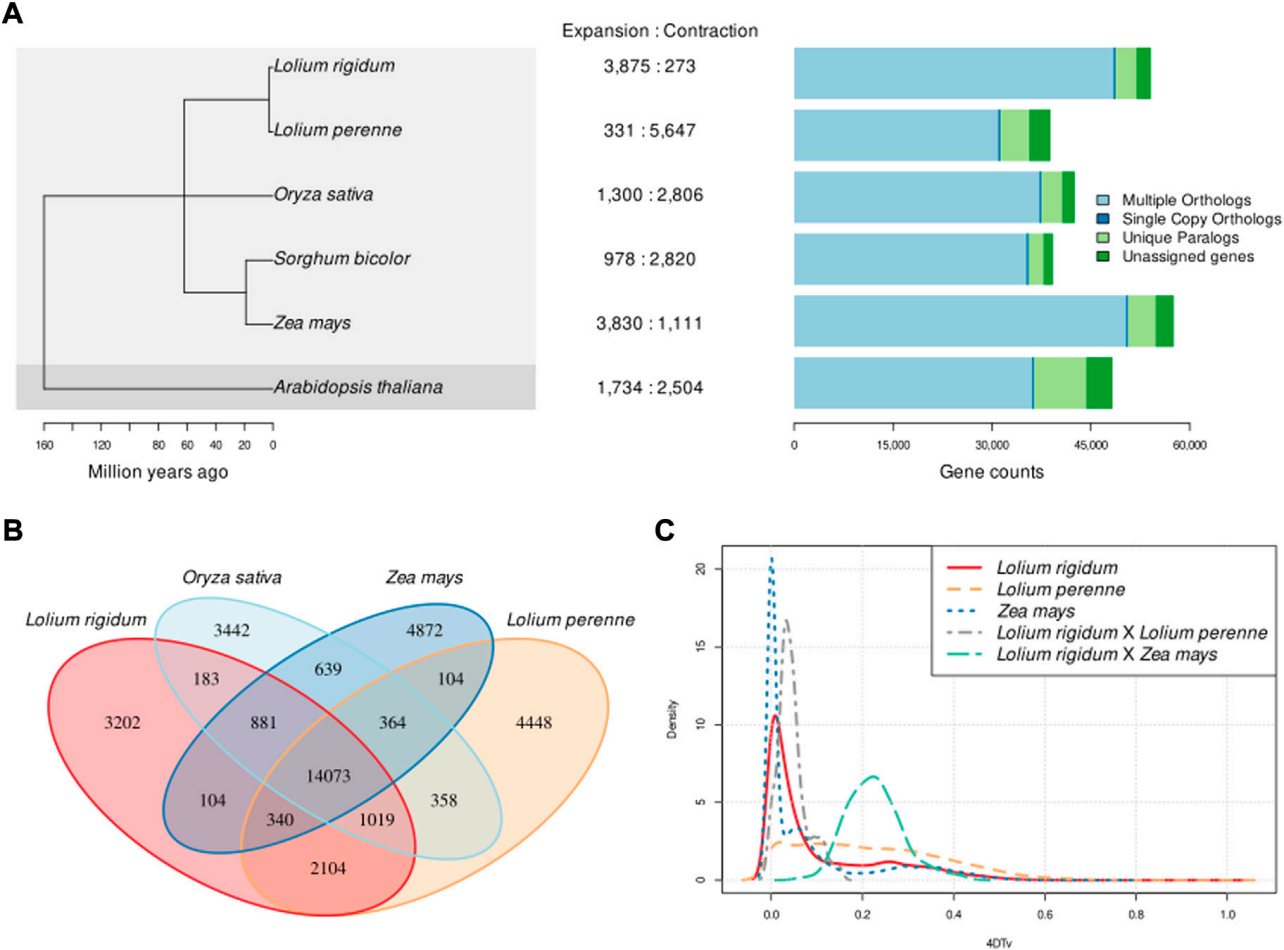
*Lolium rigidum* comparative genomics. (**A)** (left): phylogeny based on single-copy gene orthologs (100% bootstrap support across all clades); (**A)** (centre): number of significantly expanded and contracted gene families; (**A)** (right): distribution of genes with multiple orthologs, single-copy orthologs and unique orthologs, **(B)** Venn diagram of shared gene families between *L. rigidum*, *L. perenne*, *O. sativa*, and *Z. mays*; **(C)** distribution of the transversion rates in four-fold degenerate sites (4DTv) within orthogroups in *L. rigidum*, *L. perenne* and *Z. mays*.

The comparison between the genomes of *L. rigidum*, *A. thaliana*, and the four grass crop species is summarised in Figure 2. We confirmed that *L. rigidum* is very closely related to *L. perenne* and that the five grass species share a common ancestor around 65 MYA (Figure 2 panel a). The four grass species, *L. rigidum*, *L. perenne*, *O. sativa,* and *Z. mays* have more shared gene families than species-specific gene families, and *L. rigidum* shares more gene families with *L. perenne* than with *O. sativa* and *Z. mays* (Figure 2B). Gene families are, on average, ∼14 times more expanded than contracted in *L. rigidum*. This is in striking opposition to the *L. perenne* genome where gene families are ∼19 times more contracted than expanded (Figure 2 panel A central area). The *S. bicolor* genome also exhibited more contraction than expansion, but this is only slight compared with that of *L. perenne*. Also surprisingly, the distribution of genes with multiple orthologs, unique paralogs, and single-copy orthologs in *L. rigidum* is more similar to that of *Z. mays* than *L. perenne*. The distribution of 4DTv in Figure 2C shows two things: first, *L. rigidum* expectedly diverged more recently from *L. perenne* than from *Z. mays*; and second, *L. rigidum* experienced a recent WGD event while *L. perenne* experienced repeated and older WGD events as evidenced by the comparatively flatter 4DTv distribution.

GO term enrichment analysis of significantly expanded gene families in *L. rigidum* reveals herbicide resistance-related biological functions are significantly enriched (*p*-value<0.05; 368 significantly enriched GO terms from 15,192 gene names). The 15 most significantly enriched GO terms are presented in Supplementary Table S3. The following five GO terms showed consistent enrichment across two independent sets of 500 randomly sampled gene names from the full set of significantly expanded gene families: cellular response to stimulus (GO:0051716), response to stimulus (GO:0050896), regulation of metabolic process (GO:0051252), regulation of biosynthetic process (GO:2001141), and organic substance metabolic process (GO:0071704).

### Herbicide resistance genes

There is statistically significant evidence for the expansion of the detoxification gene families tested, except for the monodehydroascorbate reductase (MDAR). Interestingly, there was no evidence for significant expansion of the EPSPS gene family. Instead, there is evidence for positive selection on one EPSPS gene, specifically at a site located between position 556 and 570 bp of the consensus CDS (Supplementary Figure S2). This position is conserved between *L. rigidum*, and *L. perenne*, but not with *A. thaliana* and *S. bicolor*. This putative target-site mutation, in addition to the expansion of detoxification genes, hints at the possible basis of glyphosate resistance in the reference genotype.

## Discussion

### Genome assembly

A combination of long- and short-read sequence assembly scaffolded with Hi-C proved to be sufficient to obtain a high-quality, chromosome-level 2.4-Gb reference genome of *L. rigidum*. The very high proportion of duplicated genes is common in plant genomes (Panchy et al., 2016) but the genome was relatively unambiguous with no evidence of polyploidisation according to the distribution of 4DTv. The simple diploid nature of annual ryegrass made it easier to resolve contig placements compared to polyploid species (Kyriakidou et al., 2018). The reference genome is mostly repetitive, consisting of long terminal repeat (LTR) families. Such expansion of LTRs in genomes has been linked to crop domestication (Qin et al., 2014; Huang et al., 2017) which annual ryegrass is, before becoming a noxious weed. Despite the high-quality of this genome assembly, additional sequencing efforts could improve it further, i.e. individual chromosome sequencing can be performed in the future.

### Comparative genomics and herbicide resistance genes

The phylogeny inferred using our assembly and the reference genomes of five other plants matched the expected relationships and divergence times. *L. rigidum* indeed diverged later from and shares more gene families with the other grass species than with *A. thaliana*. Despite being closely related and having similar genome sizes, the patterns of gene expansion and contraction in *L. rigidum* and *L. perenne* are the opposite of each other. This suggests that *L. rigidum* underwent recent single-gene duplication events which is further supported by the distribution of 4DTv. These single-gene duplication events may have been mediated by tandem duplication (gene duplication resulting in multiple paralogous genes adjacent to each other) which is supported by the proximity of the expanded detoxification genes.

The expansion of herbicide resistance-related gene families is another interesting finding, with six out of the seven detoxification gene families tested showing significant expansion. This, in conjunction with evidence for positive selection in one of the EPSPS genes without expansion of the whole family, suggests that the mechanism of glyphosate resistance in this specific plant genotype is already multifactorial. Glyphosate resistance here is likely achieved through a combination of intensified neutralisation of reactive oxygen species (ROS) by the increased number of detoxification enzymes, and possibly by rendering the EPSPS enzyme resistant to disruption by glyphosate molecules. Given that we have stronger evidence for the former rather than the latter, we suggest that ROS scavenging by detoxification genes may be more important than preventing the disruption of EPSPS activity in aromatic amino acid synthesis in the reference genotype sequenced.

## Conclusion

We have assembled the first reference genome of the agriculturally important and noxious weed species, *Lolium rigidum*, at a high-quality and at chromosome level. This reference genome is pivotal in deciphering the genetic bases of new and emerging herbicide resistances, and the development of modern molecular tools for the management of this highly herbicide-resistant weed species. Upon analysing this reference genome representing only a single genotype, we were able to gather some evidence for the multifactorial bases of glyphosate resistance, i.e. target site resistance conferred by single point mutations within the gene, and non-target site resistance through the extensive duplication of detoxification genes. Hence, it is doubtless that this reference genome will be crucial for the genetic mapping of herbicide resistance, making use of more genotypes in more sophisticated experimental designs. It will also be instrumental in the development of new and novel genomically informed weed and herbicide resistance control strategies including genomic prediction models which will improve the speed and cost-effectiveness of herbicide resistance assays.

## Data Availability

The datasets presented in this study can be found in online repositories. The names of the repository/repositories and accession number(s) can be found in the article/Supplementary Material.

## Supplementary material

SUPPLEMENTARY FIGURE S1 Panel A: Lolium rigidum plant. Panel B: Hi-C interaction heatmap highlighting the 7 Lolium rigidum chromosomes. The unlabelled partition at the bottom-right corner of the heatmap refers to 1,757 unplaced contigs with some correlation with other regions coinciding with high repeat contents.

SUPPLEMENTARY FIGURE S2 Distribution of long-terminal repeat retrotransposon assembly index (LAI; measure of the intactness of these LTR retrotransposons (LTR-RTs) which is proportional to the contiguity of the assembly) across the Lolium rigidum genome assembly.

SUPPLEMENTARY FIGURE S3 Ka/Ks (ratio of the number of nonsynonymous substitutions per non-synonymous site to the number of synonymous substitutions per synonymous site per unit time) across non-overlapping 15-bp sliding windows comparing an enolpyruvylshikimate phosphate synthase (EPSPS) gene of L. rigidum (i.e. XP_047075216.1) to the EPSPS genes of O. sativa, S. bicolor, a paralog in L. rigidum, and two homologs in A. thaliana. Red asterisks show significant peaks at *p*≤0.001.
