# Supplementary figures and images for: Rounding up the annual ryegrass genome: High-quality reference genome of Lolium rigidum
Source: Front Genet. 2022 Nov 1;13:1012694. doi: 10.3389/fgene.2022.1012694 (PMC9664059; doi:10.3389/fgene.2022.1012694)

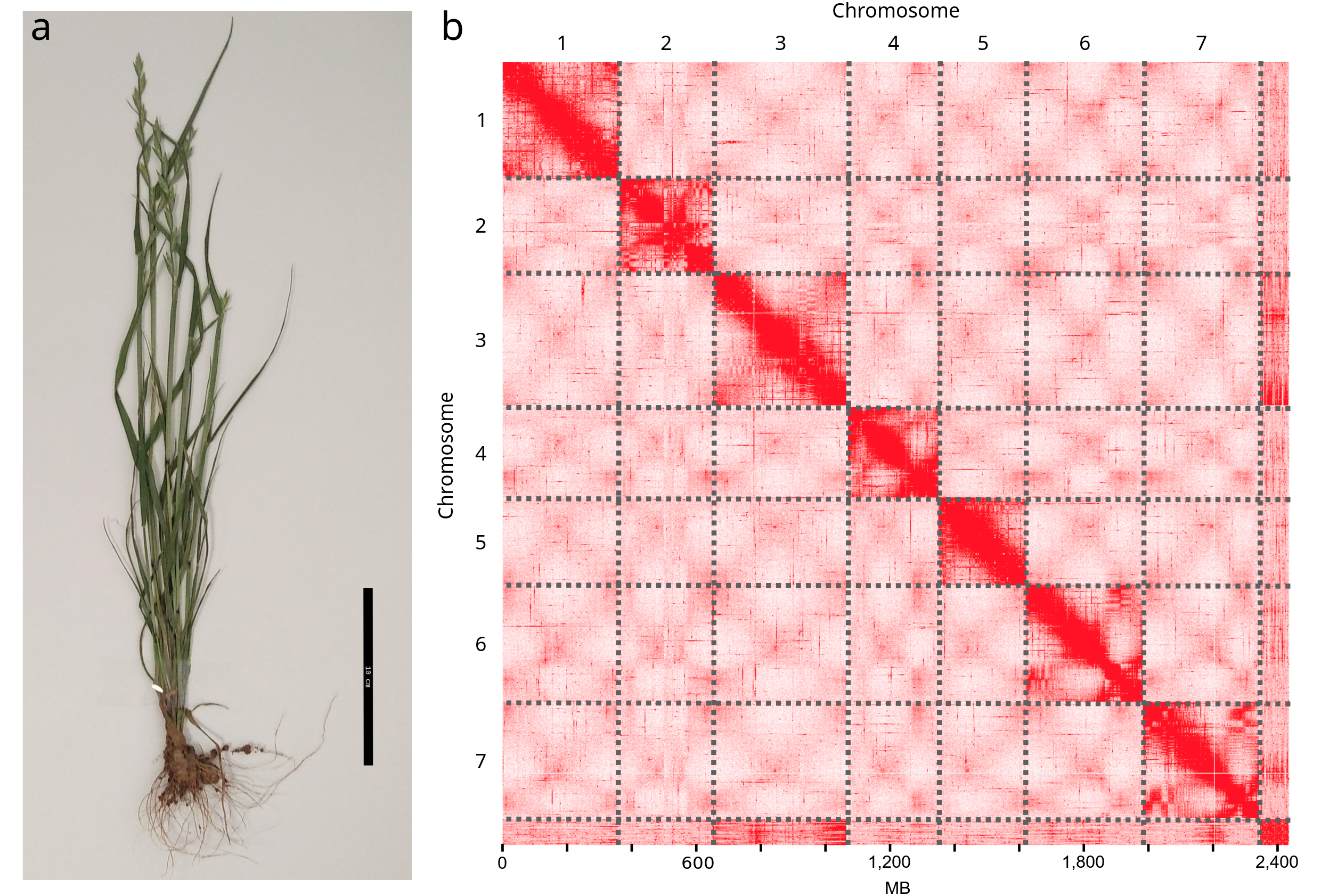

Supplement: Supplementary file 1 [file Presentation1.zip › Supplementary Figure S1.tiff]

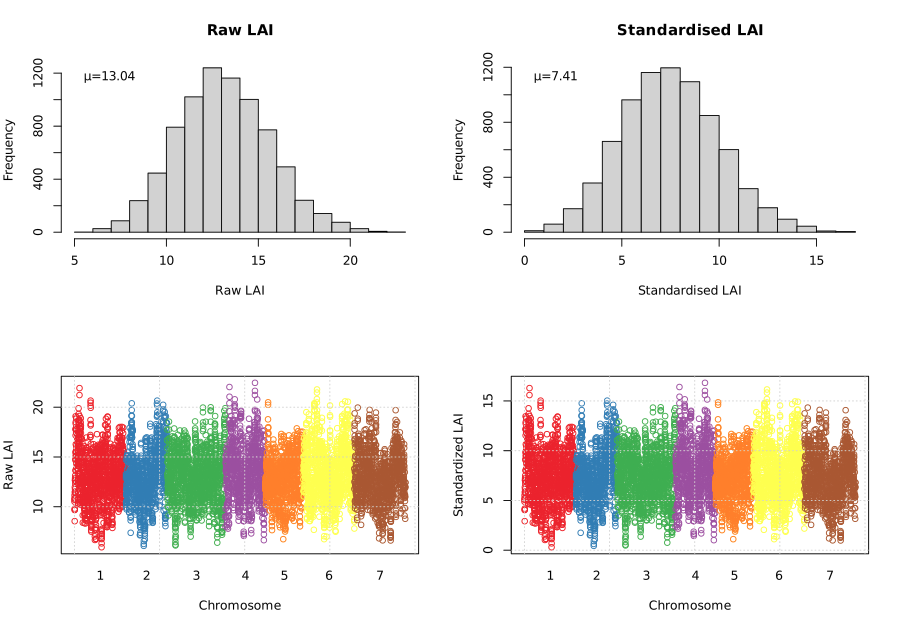

Supplement: Supplementary file 1 [file Presentation1.zip › Supplementary Figure S2.tiff]

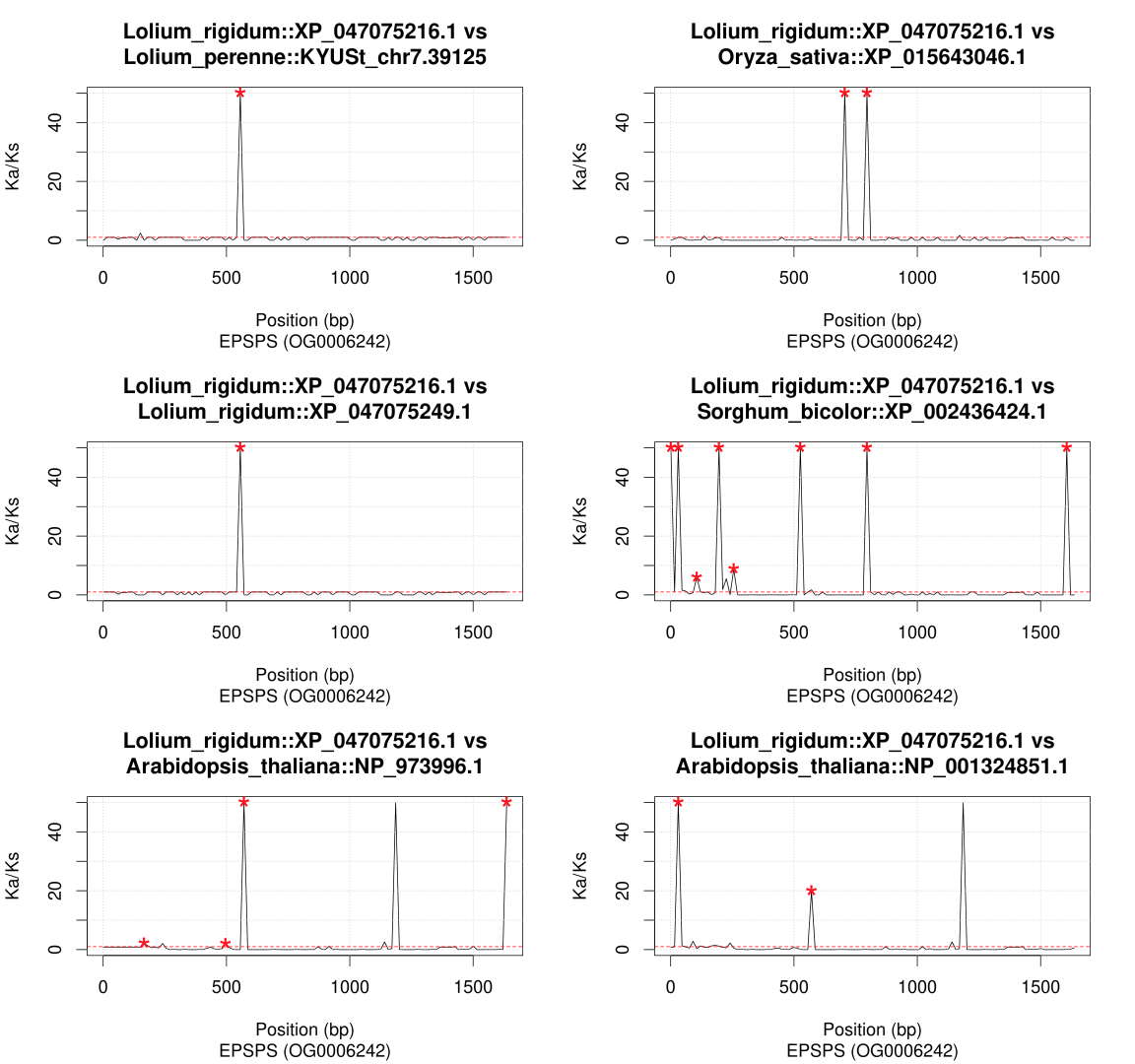

Supplement: Supplementary file 1 [file Presentation1.zip › Supplementary Figure S3.tiff]
